# Differences in resistance mutations among HIV-1 non-subtype B infections: a systematic review of evidence (1996–2008)

**DOI:** 10.1186/1758-2652-12-11

**Published:** 2009-06-30

**Authors:** Jorge L Martinez-Cajas, Nitika P Pai, Marina B Klein, Mark A Wainberg

**Affiliations:** 1 Department of Medicine, Infectious Diseases, Queen's University, Kingston, Ontario, Canada; 2 McGill University Health Centre, Montreal, Quebec, Canada; 3 McGill University AIDS Centre, Jewish General Hospital, Montreal, Quebec, Canada

## Abstract

Ninety percent of HIV-1-infected people worldwide harbour non-subtype B variants of HIV-1. Yet knowledge of resistance mutations in non-B HIV-1 and their clinical relevance is limited. Although a few reviews, editorials and perspectives have been published alluding to this lack of data among non-B subtypes, no systematic review has been performed to date.

With this in mind, we conducted a systematic review (1996–2008) of all published studies performed on the basis of non-subtype B HIV-1 infections treated with antiretroviral drugs that reported genotype resistance tests. Using an established search string, 50 studies were deemed relevant for this review.

These studies reported genotyping data from non-B HIV-1 infections that had been treated with either reverse transcriptase inhibitors or protease inhibitors. While most major resistance mutations in subtype B were also found in non-B subtypes, a few novel mutations in non-B subtypes were recognized. The main differences are reflected in the discoveries that: (i) the non-nucleoside reverse transcriptase inhibitor resistance mutation, V106M, has been seen in subtype C and CRF01_AE, but not in subtype B, (ii) the protease inhibitor mutations L89I/V have been reported in C, F and G subtypes, but not in B, (iii) a nelfinavir selected non-D30N containing pathway predominated in CRF01_AE and CRF02_AG, while the emergence of D30N is favoured in subtypes B and D, (iv) studies on thymidine analog-treated subtype C infections from South Africa, Botswana and Malawi have reported a higher frequency of the K65R resistance mutation than that typically seen with subtype B.

Additionally, some substitutions that seem to impact non-B viruses differentially are: reverse transcriptase mutations G196E, A98G/S, and V75M; and protease mutations M89I/V and I93L.

Polymorphisms that were common in non-B subtypes and that may contribute to resistance tended to persist or become more frequent after drug exposure. Some, but not all, are recognized as minor resistance mutations in B subtypes. These observed differences in resistance pathways may impact cross-resistance and the selection of second-line regimens with protease inhibitors. Attention to newer drug combinations, as well as baseline genotyping of non-B isolates, in well-designed longitudinal studies with long duration of follow up are needed.

## Background

The vast majority of cases of HIV infection worldwide are due to non-subtype B HIV-1 [1]. The HIV-1 group M has been classified into subtypes, as well as circulating and unique recombinant forms (CRF and URF respectively), because of significant natural genetic variation. This diversification includes subtypes A to K and many CRFs and URFs.

Subtype B is the most prevalent in the western world (western Europe, the Americas, Japan and Australia), while non-B subtypes predominate in the rest of the world: subtype C in sub-Saharan Africa and India; CRF01_AE in South-East Asia; CRF02_AG in west Africa,;and subtype A in eastern Europe and northern Asia [1]. In addition, the proportion of non-B subtypes in North and South America and western Europe is increasing [2-6]. Thus, it is expected that non-B subtypes will become more common in the western world over time.

Combination antiretroviral therapy (ART) is now used in many areas of the world, and HIV resistance to antiretroviral drugs (ARVs) has emerged in all locales. Resistance to ARVs in non-B subtypes is less well studied than in subtype B, mainly because of the predominance of subtype B in those countries in which ARVs first became available.

Yet there is clearly a potential for genetic differences among subtypes to yield differential patterns of resistance-conferring mutations in response to ARV pressure. This possibility is supported by the finding that HIV-1 naturally varies in genetic content among subtypes by as much as 35% [7].

Because differences in codon sequences at positions associated with drug resistance mutations might predispose viruses of different subtypes to encode different amino acid substitutions, it is possible that HIV-1 genetic diversity might influence the type of resistance mutations that emerge upon drug exposure, as well as the rate of emergence of resistance. It is further conceivable that this diversity could affect the degree of cross-resistance to ARVs within a drug class. The result could impact clinical outcomes (i.e., virologic suppression and/or preservation of immunologic function).

As an example, data from studies on the use of single dose nevirapine (sdNVP) for prevention of mother to child transmission (PMTCT) have revealed that subtype C is more prone to acquire nevirapine (NVP) resistance mutations than either subtype A or D, and this can reduce subsequent responsiveness to antiretroviral therapy [8]. Similarly, virological and biochemical data suggest that amino acid background naturally present in target proteins might affect the magnitude of resistance conferred by typical antiretroviral resistance mutations [9].

On the other hand, studies on antiretroviral drug resistance in non-B HIV-1 subtypes exposed to chronic suppressive therapy have yielded less definitive results with respect to the importance of natural HIV-1 genetic diversity in regard to acquisition of drug resistance mutations.

Genotypic ARV resistance data is useful in deciding on best choice of ARVs for individual treatment and provides a repository of information on the presence of HIV resistance mutations among non-B subtypes. Because resistance mutations among HIV-1 subtypes may vary, lack of information on specific resistance mutations in non-B subtypes may result in non-detection of clinically important resistance or misinterpretation of resistance in such subtypes.

Although HIV resistance databases make efforts to incorporate newer genotypic data into their pools of data, the availability of HIV genotypes from areas of the world with non-B subtype predominance is remarkably low compared to that of subtype B [10]. The reasons for this scarcity of data are probably related to reduced availability of ARV therapy, the high cost of drug resistance testing, and a paucity of research facilities in resource-limited countries.

Treatment decisions are often based on CD4 cell quantification or clinical signs of therapeutic failure. Viral load testing is not regularly conducted and resistance testing may only be performed for participants enrolled in study cohorts or trials, but not as a matter of general practice. Hence, a heightened interest in studying HIV resistance in such countries in the context of programmes for expanded access to ART does not come as a surprise.

Editorials, perspectives and narrative reviews have been published on several aspects of non-B HIV subtypes, yet a systematic review has not been executed. With this in mind, we conducted a systematic review of all published and unpublished literature on all aspects of non-B subtypes, i.e, genetic and biochemical diversity, resistance, disease progression, transmission and PMTCT.

For this review, the second in the series, we included data from studies that reported resistance in non-B patients failing ART. Our primary objective was to synthesize available knowledge on resistance to ARV in non-B subtype HIV-1 patients taking chronic, suppressive ART. Our secondary objectives were to: elicit differences in resistance mutations within non-B HIV-1 subtypes; identify knowledge gaps; and delineate future research required to fill such gaps.

## Methods

### Identification of studies

#### Search string, key words and search terms

Our search string included key words and search terms as follows: Search #1: "HIV"[11] OR "HIV-1"[11] OR "HIV-1". Search #2: "non-b" [TIAB] OR "subtype*" [TI] OR "clade" [TI] OR "strain*" [TI] OR "variant*" [TI] OR "non-subtype B*" [TIAB]. When performing the search in databases that did not accept MeSH terms, we used key words, such as "subtype", "CRF", "clade", "resistance", "mutations" and "HIV-1 isolates".

#### Timeframe

Our search strategy covered the period between January 1996 and November 2008. Our search was limited to publications in English.

#### Study selection

**Figure 1:**
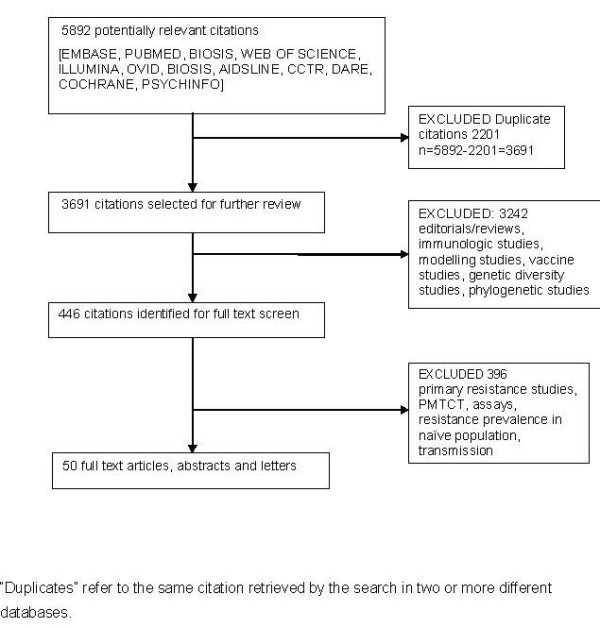
**Flow diagramme for study selection**.

The study selection methodology is shown in Figure 1. We searched 11 electronic databases of full-text articles and conference abstracts, i.e., PUBMED (1996–2008), Web of Science (1996–2008), EMBASE (1996–2008), BIOSIS (1996–2008), AIDSLINE (1996–2005), OVID (1996–2008), Psychinfor (1996–2008), Cochrane controlled trials register (1996–2008), DARE (1996–2008), COCHRANE (1996–2008), and ILLUMINA (1996–2007). A total of 5892 references were identified from these 11 databases. After excluding duplicates (the same reference found by two database engines), 3691 citations were considered relevant after the first screen.

We then divided these citations into several groups, i.e., genetic and biochemical diversity, disease progression, PMTCT and transmission studies. On the first screen, we reviewed the titles of the articles, and if the title was clearly not related to the topics at hand, the reference was removed; otherwise it was kept for the second screen (446 citations). In this screen, abstracts were examined and articles whose content was clearly unrelated to our focus were removed. If the abstract did not provide enough information to support an inclusion or exclusion decision, the full article was reviewed. After this process, only 50 articles and abstracts were found relevant.

### Inclusion criteria

We included full text articles, abstracts and letters, provided that they contained relevant information and were of sufficient completeness. Conference abstracts were searched. These were: Conference on Retroviruses and Opportunistic Infections (1997–2008), Interscience Conference of Antimicrobial Agents and Chemotherapy (2000–2008), IAS (2000–2008), Infectious Diseases Society of America (2000–2008), and International HIV Drug Resistance Workshop (2000–2008]). We also searched bibliographies and references from primary studies and review articles.

Included studies either: identified mutations selected in non-B subtype ART recipients; reported non-B subtype infections identified by clear geographical predominance or by direct phylogenetic analysis of the genotyped samples; determined statistical associations of mutations and therapies; compared relative frequency of resistance mutations shared by B and non-B subtypes; or examined differences in frequencies among subtypes of polymorphisms both before and after drug exposure.

### Exclusion criteria

Excluded studies either: did not report drug resistance; reported only biochemical differences between subtypes in regard to the reverse transcriptase and protease enzymes; reported the prevalence of resistance mutations only in ART-naïve patients (n = 115); reported only genetic diversity; or reported only on transmission of non-B subtypes.

### Data abstraction

The final data abstraction was independently performed by two reviewers (JLM, NPP). While one reviewer (JLM) abstracted all the studies, the second reviewer (NPP) extracted data from a subset (25%). Inter-rate agreement between reviewers was calculated using kappa statistics and was high (> 90%). Discordant opinions were resolved by further review and discussion of the articles until an agreement was reached.

The data abstraction form included the following general components: name or names of authors, year of publication, study location, study design, sample size, HIV-1 subtypes (details), ART (details), genotyping techniques, and criteria to define resistance mutations.

We also assessed: the clarity of the research question; whether therapeutic failure occurred during first-line therapy or later; if there were analyses of mutations according to use of relevant drugs or drug combinations; the representativeness of genotyped isolates; type of sampling; if there were comparisons among HIV (B or non-B) subtypes; and if genotypes were compared with consensus sequences derived from pre-therapy isolates (Table S1; Additional File 1).

Furthermore, data were also collected on: detection of novel resistance mutations; measurement of frequency of resistance mutations before and after ARV exposure; and details of the relationships of such mutations to a particular drug or relevant drug combination as well as HIV-1 subtype.

## Results

The final 50 study sub-set has been tabulated (Table S2; Additional File 2) [12-61]. Although a vast majority (86%) of the studies were observational, a small proportion (4%) were randomized controlled trials.

### Primary finding

Overall, similar mutations were present in both B and non-B subtypes. However, some differences in the types and frequencies of resistance mutations were reported and summarized (Table S3; Additional File 3). A synthesis of study findings with respect to three major ARV drug classes is listed here:

#### Findings with respect to NRTI resistance

First, in subtype C-infected patients treated with the NRTI backbone ZDV/ddI in Botswana, a different thymidine analogue resistance pathway (67N/70R/215Y) was observed and reported [43]. Yet this was not reported in studies from subtype C patients on similar therapy in India, South Africa or Malawi [16,24,33,41].

Second, the incidence of K65R was geographically differentiated in subtype C. A study from Bostwana reported a high incidence of K65R (30%) in subtype C patients who received d4T/ddI + NVP or EFV [26]. Another study from Malawi detected either K65R or K70E in 23% of patients failing first-line therapy with d4T/3TC/NVP [33]. A study from South Africa, meanwhile, detected K65R in 7% and 15% of patients failing first-line or second-line regimens whose nucleoside backbones included d4T/3TC or ddI/ZDV [56]. In contrast, other studies from India, Israel, South Africa and Botswana did not report high frequencies of K65R in subtype C viruses [16,24,29,41,46,50]. K65R also seems to be less frequent in subtype A than in all other subtypes, despite use of similar treatment regimens [32].

Third, in subtype C isolates from India, the pre-treatment and post-treatment frequencies of mutations E203D/K/V/N/A (2% vs 24.7%), H208Y (0% vs 14.7%) and H221Y (0 vs 13.7%) suggest a likely role of these mutations in NRTI resistance [24] and extend previous observations with subtype B. The degree of statistical association in the study from India was very strong after exposure to one cycle of NRTIs, while it was evidently weaker, yet statistically significant, in the B subtype study after exposure to one or two nucleoside reverse transcriptase inhibitors (NRTIs) [62].

In subtype F from Brazil, a preference for mutations at position 211 rather than position 210 was seen in NRTI-resistant isolates [15]. Finally, a higher propensity to acquire thymidine analog-associated mutations (TAMs) was reported in patients carrying CRF_06cpx (AGK recombinants) compared to patients carrying CRF02_AG from Burkina Faso [53].

#### Findings with respect to NNRTI resistance

With regard to non-nucleoside reverse transcriptase inhibitor (NNRTI) resistance, the importance of the V106M mutation in non-B subtypes has been confirmed in recent years. Six studies confirmed that V106M is frequently seen in non-B subtypes (C and CRF02_AE) after therapy with EFV or NVP [24,29,34,43,49,50].

The G190A mutation was also relatively more frequent among subtype C-infected patients failing NNRTI-based therapy in Israel and India. In the Israeli, but not the Indian study, G190A/S was seen as a natural polymorphism in subtype C [24,29]. In both studies, the frequencies of these mutations among treated patients were higher than in subtype B and C drug-naïve patients.

A large survey reported that reverse transcriptase (RT) residues 35 in subtype A, 98 and 106 in subtype C, 35 and 98 in subtype G, as well as 98 in CRF02_AG, were more frequently mutated than was the case in subtype B [38]. In the same study, other RT positions were less frequently mutated in other subtypes than in subtype B after exposure to ARVs as follows: RT residues 39 and 179 in subtype A; residues 35, 48, 121 and 166 in subtype C; residue 39 in subtype D; residue 39 in subtype F; residues 39 and 104 in subtype G; residue 162 and 238 in CRF01_AE; and residue 39 in CRF02_AG [38].

#### Findings with respect to HIV protease mutations

In the two studies that assessed protease (PR) mutations in patients failing NFV therapy, the D30N mutation was never observed in either CRF02_AG or CRF02_AE isolates. Rather, the 88S mutation emerged after NFV use in CRF02_AE and after IDV use in subtype B [17,21]. A third study also reported an absence of the D30N mutation in CRF02_AE patients receiving protease inhibitors (PIs), including NFV, but no information on the specific PI used by the patients was available [51].

A low frequency of D30N was seen in subtype C isolates from Israel after NFV usage versus a higher frequency in subtype C viruses from Botswana [25,30], suggesting that subtype C viruses from Ethiopia (the origin of the Israeli samples) and southern Africa might behave differently.

The M89I/V mutations have been identified in F, G and C but not other subtypes [59]. The V82M mutation was found to emerge in subtype G, but not in B [13]. Finally, the L90M mutation is rare in subtype F, but common in subtype B from Brazil [18].

Kantor et al reported that positions more frequently mutated in PR in non-B subtypes included residues 14 in subtype A, 13 and 64 in subtype C, 37 and 65 in subtype F, 71 in subtype G, 62 and 64 in CRF01_AE, and 15 and 71 in CRF02_AG [40].

Additionally, positions that were less frequently mutated in non-B subtypes after exposure to ARVs include changes at PR residues 10, 20 and 63 in subtype A, residues 20, 53, 63, 74 and 82 in subtype C, residues 13 and 20 in subtype D, residues 10, 14, 20 and 77 in subtype F, residues 20, 67, 73, 82 and 88 in subtype G, residues 20, 63, 82 and 89 in CRF01_AE, and residue 20 in CRF02_AG [41]. Another study identified a possible role of mutations at positions 13, 16, 33, 37, 41, 57, 65, 72, 74 and 89 in resistance to PIs [58].

### Rate of acquisition of resistance mutations

Only one study compared the rate of accumulation of resistance mutations between patients infected with subtypes B versus C, and revealed higher rates of emergence of NRTI and PI resistance mutations and equal rates of emergence of NNRTI mutations in subtype B compared to C [48]. Although retrospective, this study measured factors that could influence the acquisition of resistance; these included CD4 cell count, viral load at initiation of ART, and time of resistance genotyping.

## Discussion

The overall findings in our review are consistent with the notion that mutations associated with resistance in B resemble those in non-B subtypes, and might therefore lead to the conclusion that HIV-1 genetic diversity bears only a slight effect on ARV-selected mutations. However, this idea is mistaken, and, there are genuine subtype differences in both the types of resistance mutations and preferred pathways of resistance.

Indeed, certain mutations may emerge almost exclusively in some non-B subtypes. This review makes it evident that the studies performed on this topic have been diverse and most were not specifically designed to assess the impact of HIV genetic diversity on resistance to ARVs in the context of chronic antiretroviral therapy. Some of the reasons for these limitations and recommendations for future studies and/or secondary analyses of available data are discussed below.

### Types and frequency of resistance mutations

Among the most important differences are: the protease mutation 82 M in subtype G versus 82A/F/S/T in the others; 88D in subtype B versus 88S in C and AG; and the RT mutation V106M in subtype C and CRF01_AE versus V106A in subtype B. Also, polymorphisms at RT residue 98, common in subtype G, are associated with NNRTI resistance in subtype B, and may lower the resistance barrier and duration of efficacy of NNRTIs [14].

The available evidence indicates that the frequency of some resistance mutations shared by B and non-B subtypes can vary after failure of first-line therapeutic regimens, as in the case of the K65R mutation. These differences in type and frequency of resistance mutations should not be underestimated vis-à-vis impact on remaining active regimens in resource-limited settings.

The 67N/70R/215Y TAM pathway reported to predominate in subtype C in Botswana will probably be adequately detected by most resistance algorithms since it does not involve new mutations. It seems, though, that such a pathway is uniquely associated with d4T/ddI exposure in subtype C since no other study in our review reported this pattern. Notably, subtype C studies from South Africa, Malawi and India, in which d4T or ZDV plus 3TC as backbone have been employed, and a study on CRF01_AE exposed to ZDV/ddI failed to report this pattern [16,24,33,39].

The finding of higher frequencies of the K65R mutation in subtype C and not other subtypes [26,33,56] suggests that subtype C viruses may enjoy a particular predisposition toward acquiring this mutation, and this has been described in vitro. A subtype C RNA template mechanism has been proposed to explain this phenomenon in which neither codon bias nor RT enzyme subtype plays a role [63,64].

However, not all studies have found a higher prevalence of K65R in subtype C, and it is possible that such discordance is related to the duration of sub-optimal therapy in patients inadvertently experiencing virological failure. All studies reporting a low frequency of K65R have monitored virologic failure, while those that found high frequencies of this mutation monitored therapeutic failure based on immunological or clinical parameters, which require several months of surveillance after virological failure and resistance are suspected [65-67].

Bias may also have been introduced by virtue of the fact that many patients began therapy with ZDV, which selects for TAMs that can in turn mitigate against the selection of K65R. The foregoing heightens the need to detect virological failure as early as possible in ART-access programmes worldwide.

### Rate of emergence of resistance

Only one study that compared rates of emergence of resistance between subtypes in patients receiving suppressive ART actually reported a lower risk of accumulation of major resistance mutations in subtype C than in B [48]. This is paradoxical, since all the ARV drugs employed were originally designed to target subtype B.

Qualitatively, the major mutations that emerged in both subtypes were the same. Viral, host and drug factors were mostly the same among participants. The authors inferred that both subtype B and C patients possessed similar profiles of virologic failure after use of the same ART regimens. Therefore other unknown factors might have been responsible for any subtype differences observed.

However, subtype C HIV-1 might not need to accumulate a similar number of B-defined major mutations to reach an equivalent level of resistance. For example, several minor resistance mutations in subtype B PR occur more frequently as natural polymorphisms in subtype C, e.g., 36I, 89M, 93L.

Thus, it is conceivable that there might be a lower accumulation threshold of major mutations in C subtypes if we assume that these natural polymorphisms act similarly in subtype C as they do when present as secondary resistance mutations in subtype B. This hypothesis requires further testing.

### Limitations of available evidence

Important heterogeneity across studies was found in terms of design, reporting, location, mutations, and comparisons. Study designs were cross-sectional, longitudinal and clinical.

Some studies were unique in design and limited in information, thus restricting the possibilities for comparison. For instance, one study evaluated codon usage in subtypes B, C and F [25]; another study surveyed the frequency of the 82 M mutation, without making reference to other mutations [19].

Also, studies were inconsistent in reporting follow up, adequate sample size, representativeness of the sample, comparisons, study designs and isolate sequences. They also lacked details on the level of ARV experience of patients, as well as clarity of objectives. Reporting of consistent measures was lacking for frequency of mutations by subtype and by specific drugs or drug combinations.

Several studies reported pooled non-B data, pooling information from several subtypes as one category. Studies have also addressed different research questions and used non-equivalent NRTI backbones, e.g., ZVD/ddI and ZDV/3TC. Several studies grouped mutations by drug class without information on the nature of the regimen at virologic failure, and have reported resistance in different ways, e.g., different algorithms or resistance lists.

Furthermore, no study in non-B subtypes reported genotyping data prior to ARV exposure. Only one paper reported having generated a baseline consensus sequence from HIV sequences obtained in the geographical region in which the study was conducted, but was limited in that the population used to generate the consensus sequence was different from the ART-exposed population [24]. Hence, only a narrative description was possible.

Not all studies could relate mutations to specific drugs. A majority of studies were conducted in patients failing second-line or third-line ART, while a small minority (five) were conducted in patients who failed first-line ART regimens [14,21,24,37,41].

In addition, few longitudinal studies evaluated resistance mutations in a particular non-B subtype and compared genotypes of viruses from treated patients on the basis of consensus sequences, i.e., of the same subtype. The result is that available knowledge on PI resistance mutations seems to originate from one or two studies that represent a very small sample of the worldwide patient population.

In addition, 22 of 50 (44%) of studies performed in individuals with non-B infections evaluated drugs or regimens no longer recommended by international guidelines, e.g., the NRTI backbones, d4T/ddI or ZDV/ddI, and the PIs, IDV and NFV [68-70].

Our review also included more recent studies from several African countries and India that evaluated first-line therapeutic regimens that employed ZDV/3TC or d4T/3TC, plus either NVP or EFV. These studies have not reported any novel mutations, but they did detect important associations of polymorphisms with ARV resistance. Deshpande et al discovered that the A98G/S substitution was strongly associated with NNRTI treatment failure in subtype C [24].

Prior to that publication, the A98G/S substitution was not considered to be important in all ARV resistance algorithms, apparently because subtype C wild type viruses independently contain A98G/S as a common polymorphism [29]. Desphande et al also observed a number of RT polymorphisms that increased in frequency after therapy at positions K20, E28, W88, V90, and V108 [21]. The A98S polymorphism is also frequently found in subtype G and might consequently contribute to resistance in that subtype.

### ARV cross-resistance in non-B subtypes

Resistance in non-B subtypes has rarely been reported on the basis of single drugs or NRTI backbones that are currently in use in Western countries. Rather, mutations have been reported for drug classes. Hence, cross-resistance can be estimated only for some NRTIs and NNRTIs, but not for most PIs that are the only drugs being used as part of second-line regimens in most regions of the world.

Hence, well-informed guidance for sequential use of PIs in populations affected by non-B subtypes is difficult to obtain. For instance, in the case of NFV, the potential for cross-resistance in viruses of CRF01_AE and CRF02_AG origin could be higher than has been observed in subtype B due to the preferential selection of the N88S and L90M mutations. Of note, NFV was the most commonly used PI in resource-limited settings until recently.

Similarly, NRTI backbones may also vary in the mutational profile that they select, based on drug combinations. Newer drugs, e.g., TDF and ATV/r are now preferred in resource-rich countries and need to be studied in non-B subtypes to determine whether they can lower risks of accumulation of resistance mutations. This is important in view of the higher propensity of subtype C to acquire K65R.

HIV resistance databases continue to enter HIV genotype data from non-B subtype variants. So far, however, very few datasets, such as the Stanford HIV resistance database and that of the ANRS, provide information for drugs that have become first-line therapy in developed countries, including tenofovir, atazanavir, and fosamprenavir, as well as newer drugs such as tipranavir, darunavir, maraviroc, etravirine and raltegravir.

### Implications for future research

The clinical and prognostic implications of the preferential emergence of some mutations in non-B viruses, as well as changes in the frequencies of these mutations, are largely unstudied and unknown. Future research on the role of polymorphisms in non-B subtypes, that increase in frequency after drug exposure and that may contribute to drug resistance, e.g., A98G/S in RT and M36I and K20I in PR, is required.

This may be particularly important in parts of Africa, in which treatment failure may exceed 40% of patients after two years [71], and in India, where resistance rates of 80% to two drug classes have been reported after failure on first-line regimens that employed NRTI/NNRTI combinations [46].

No study has yet tested the degree of resistance or cross-resistance that certain mutational combinations (67N/70R/215Y) may confer in vitro. It is similarly important that future studies assess pre-treatment and post-treatment genotypes in order to detect associations of certain polymorphisms with drug resistance, including variations of polymorphisms in viruses of the same subtype that are located in different geographical areas. This might improve the appropriateness of selection of certain drugs over others in the context of second-line or third-line therapeutic options.

In order to better recognize inter-subtype differences, more longitudinal studies on the response to first-line ART combinations, or first-time exposure to a new drug, are needed. In these studies, it would be advisable that pre-therapy and post-therapy genotype resistance testing be performed, and that equivalent and newer drug combinations be examined.

Because clinical trials are difficult to execute in resource-limited settings, analysis of longitudinal data of this type might be the only way to estimate the possible potential advantages of one combination over others. Current data cannot ascertain whether or not HIV subtype is a factor protecting against or predisposing to therapeutic failure, and what therapeutic options are best and/or acceptable in subsequent salvage settings.

The generation of ARV resistance data for subtype B has been possible because most clinical trials have been performed in populations carrying such viruses. By contrast, only two clinical trials identified in this review provided sequencing data of non-subtype B viruses, and the NRTI combinations used in these studies are now considered to be sub-standard [68,72,73].

As a result, only NFV has been well studied in non-B subtypes [74-76], while very few data have been published on other PIs in this context. Therefore, discrepancies among HIV resistance interpretative algorithms for resistance testing may not be quickly resolved.

### Limitations and strengths of this review

We limited our review to RT and PR inhibitors and did not examine other drug classes currently in clinical use. Our review is subject to reporting and publication bias. We evaluated publications in the English language only.

However, we tried to minimize publication bias by performing a broad search that included multiple databases, conferences and abstracts. We were unable to retrieve literature from developing countries and resource-limited settings reported in languages other than English. This may affect the epidemiological strength of our conclusions. Owing to the presence of significant heterogeneity in reporting of outcomes, we could not pool data.

Nevertheless, our review has several redeeming factors in that it followed a written protocol, conducted a thorough search to identify relevant studies, and contacted authors to obtain articles not found through conventional library resources. We also attempted to reduce publication bias by including published studies, abstracts, letters, and brief reports.

## Conclusion

Our results suggest that the majority of ARV resistance mutations will be shared by viruses of all subtypes. Mutations conferring resistance to NRTIs and NNRTIs are the most similar among different subtypes. However, no clinical study has yet reported mutational patterns for PIs among non-subtype B viruses or compared newer PIs, e.g., atazanavir, lopinavir, amprenavir and darunavir, with older drugs.

Therefore, our understanding of the impact of HIV-1 genetic diversity on ARV drug resistance is incomplete and the effect on clinical outcomes will be difficult to measure in the context of chronic suppressive ART.

There is a need to more fully understand the role of HIV-1 natural and post-ARV exposure genetic variation so as to inform on the optimal use of limited ARV options in most of the world. Better recording of clinical factors and longer follow up of patients will be required to determine whether novel mutations might confer cross-resistance more efficiently in certain subtypes than others.

Future studies need to be performed longitudinally to include pre-therapy genotyping, and to report results not only on the basis of drug class, but also in a context of the NRTI backbones that were used. It will also be important to know whether certain drugs or drug classes were being employed for the first time in the patients being treated.

## Competing interests

The authors declare that they have no competing interests.

## Authors' contributions

JLMC and NPP performed the search, assessed and synthesized the data, and wrote the manuscript. MAW and MBK contributed to the preparation of the manuscript. All authors have read and approved the final manuscript.

## Supplementary Material

Additional file 1 **Table S1**. Quality score of reviewed studies. Click here for file

Additional file 2 **Table S2**. Characteristics of the studies evaluated. Click here for file

Additional File 3 **Table S3**. Major differences in subtype B versus non-B resistance genotype patterns in patients on ART. Click here for file

## References

[B1] Arien KK Vanham G Arts EJ Is HIV-1 evolving to a less virulent form in humans? Nat Rev Microbiol 2007 12 141 51 10.1038/nrmicro1594 17203103 PMC7097722

[B2] Brennan CA Brites C Bodelle P Golden A Hackett J Jr Holzmayer V Swanson P Vallari A Yamaguchi J Devare S Pedroso C Ramos A Badaro R HIV-1 strains identified in Brazilian blood donors: significant prevalence of B/F1 recombinants AIDS Res Hum Retroviruses 2007 12 1434 41 10.1089/aid.2007.0121 18184087

[B3] Locateli D Stoco PH De Queiroz AT Alcantara LC Ferreira LG Zanetti CR Rodrigues R Grisard EC Pinto AR Molecular epidemiology of HIV-1 in Santa Catarina State confirms increases of subtype C in Southern Brazil J Med Virol 2007 12 1455 63 10.1002/jmv.20955 17705166

[B4] Holguin A De Mulder M Yebra G Lopez M Soriano V Increase of non-B subtypes and recombinants among newly diagnosed HIV-1 native Spaniards and immigrants in Spain Curr HIV Res 2008 12 327 34 10.2174/157016208785132455 18691031

[B5] Descamps D Chaix ML Andre P Brodard V Cottalorda J Deveau C Harzic M Ingrand D Izopet J Kohli E Masquelier B Mouajjah S Palmer P Pellegrin I Plantier JC Poggi C Rogez S Ruffault A Schneider V Signori-Schmuck A Tamalet C Wirden M Rouzioux C Brun-Vezinet F Meyer L Costagliola D French national sentinel survey of antiretroviral drug resistance in patients with HIV-1 primary infection and in antiretroviral-naive chronically infected patients in 2001–2002 J Acquir Immune Defic Syndr 2005 12 545 52 10.1097/01.qai.0000155201.51232.2e 15793364

[B6] Njihia J Rank C Remis RS Shah L Swantee C Sandstrom P Brooks J Jayaraman G Archibald C High Proportion of Non-B Viral Subtypes Among Persons With Hiv-1 in Ontario, 2003–2005 CAHR meeting: Toronto, Canada 2007

[B7] Hemelaar J Gouws E Ghys PD Osmanov S Global and regional distribution of HIV-1 genetic subtypes and recombinants in 2004 AIDS 2006 12 W13 23 10.1097/01.aids.0000247564.73009.bc 17053344

[B8] Eshleman SH Hoover DR Chen S Hudelson SE Guay LA Mwatha A Fiscus SA Mmiro F Musoke P Jackson JB Kumwenda N Taha T Nevirapine (NVP) resistance in women with HIV-1 subtype C, compared with subtypes A and D, after the administration of single-dose NVP J Infect Dis 2005 12 30 6 10.1086/430764 15942891

[B9] Martinez-Cajas JL Pant-Pai N Klein MB Wainberg MA Role of genetic diversity amongst HIV-1 non-B subtypes in drug resistance: a systematic review of virologic and biochemical evidence AIDS Rev 2008 12 212 23 19092977

[B10] Rhee SY Kantor R Katzenstein DA Camacho R Morris L Sirivichayakul S Jorgensen L Brigido LF Schapiro JM Shafer RW HIV-1 pol mutation frequency by subtype and treatment experience: extension of the HIVseq program to seven non-B subtypes AIDS 2006 12 643 51 10.1097/01.aids.0000216363.36786.2b 16514293 PMC2551321

[B11] Cassol E Page T Mosam A Friedland G Jack C Lalloo U Kopetka J Patterson B Esterhuizen T Coovadia HM Therapeutic response of HIV-1 subtype C in African patients coinfected with either Mycobacterium tuberculosis or human herpesvirus-8 J Infect Dis 2005 12 324 332 10.1086/427337 15633090

[B12] Tupinambas U Aleixo A Greco D HIV-1 genotypes related to failure of nelfinavir as the first protease inhibitor treatment Brazilian Journal of Infectious Diseases 2005 12 324 329 10.1590/S1413-86702005000400009 16270125

[B13] Dumans AT Soares MA Machado ES Hue S Brindeiro RM Pillay D Tanuri A Synonymous genetic polymorphisms within Brazilian human immunodefidency virus type 1 subtypes may influence mutational routes to drug resistance J Infect Dis 2004 12 1232 1238 10.1086/382483 15031792

[B14] Sylla M Chamberland A Boileau C Traore HA Ag-Aboubacrine S Cisse M Koala S Drabo J Diallo I Niamba P Tremblay-Sher D Machouf N Rashed S Nickle DC Nguyen VK Tremblay CL Characterization of drug resistance in antiretroviral-treated patients infected with HIV-1 CRF02_AG and AGK subtypes in Mali and Burkina Faso Antivir Ther 2008 12 141 8 18389909

[B15] Cavalcanti AM Lacerda HR Brito AM Pereira S Medeiros D Oliveira S Antiretroviral resistance in individuals presenting therapeutic failure and subtypes of the human immunodeficiency virus type 1 in the Northeast Region of Brazil Mem Inst Oswaldo Cruz 2007 12 785 92 10.1590/S0074-02762007005000109 17992369

[B16] Barth RE Wensing AM Tempelman HA Moraba R Schuurman R Hoepelman AI Rapid accumulation of nonnucleoside reverse transcriptase inhibitor-associated resistance: evidence of transmitted resistance in rural South Africa AIDS 2008 12 2210 2 10.1097/QAD.0b013e328313bf87 18832885

[B17] Ariyoshi K Matsuda M Miura H Tateishi S Yamada K Sugiura W Patterns of point mutations associated with antiretroviral drug treatment failure in CRF01_AE (subtype E) infection differ from subtype B infection J Acquir Immune Defic Syndr 2003 12 336 42 12843744 10.1097/00126334-200307010-00007

[B18] Calazans A Brindeiro R Brindeiro P Verli H Arruda MB Gonzalez LMF Guimaraes JA Diaz RS Antunes OAC Tanuri A Low accumulation of L90M in protease from subtype FHIV-1 with resistance to protease inhibitors is caused by the L89M polymorphism J Infect Dis 2005 12 1961 70 10.1086/430002 15871131

[B19] Camacho R Godinho A Gomes P Abecasis A Vandamme A-M Palma C Carvalho A Cabanas J Gonçalves J Different substitutions under drug pressure at protease codon 82 in HIV-1 subtype G compared to subtype B infected individuals including a novel I82M resistance mutation [abstract] Antivir Ther 2005 12 Suppl 1 s151

[B20] Cane PA De Ruiter A Rice P Wiselka M Fox R Pillay D Resistance-associated mutations in the human immunodeficiency virus type 1 subtype C protease gene from treated and untreated patients in the United Kingdom J Clin Microbiol 2001 12 2652 4 10.1128/JCM.39.7.2652-2654.2001 11427587 PMC88203

[B21] Chaix ML Rouet F Kouakoussui KA Laguide R Fassinou P Montcho C Blanche S Rouzioux C Msellati P Genotypic human immunodeficiency virus type 1 drug resistance in highly active antiretroviral therapy-treated children in Abidjan, Cote d'Ivoire Pediatr Infect Dis J 2005 12 1072 6 10.1097/01.inf.0000190413.88671.92 16371868

[B22] Couto-Fernandez JC Silva-de-Jesus C Veloso VG Rachid M Gracie RS Chequer-Fernandez SL Oliveira SM Arakaki-Sanchez D Chequer PJ Morgado MG Human immunodeficiency virus type 1 (HIV-1) genotyping in Rio de Janeiro, Brazil: assessing subtype and drug-resistance associated mutations in HIV-1 infected individuals failing highly active antiretroviral therapy Mem Inst Oswaldo Cruz 2005 12 73 8 10.1590/S0074-02762005000100014 15867968

[B23] De Sa-Filho DJ Soares Mda S Candido V Gagliani LH Cavaliere E Diaz RS Caseiro MM HIV type 1 pol gene diversity and antiretroviral drug resistance mutations in Santos, Brazil AIDS Res Hum Retroviruses 2008 12 347 53 10.1089/aid.2007.0203 18327988

[B24] Deshpande A Jauvin V Magnin N Pinson P Faure M Masquelier B Aurillac-Lavignolle V Fleury HJ Resistance mutations in subtype C HIV type 1 isolates from Indian patients of Mumbai receiving NRTIs plus NNRTIs and experiencing a treatment failure: resistance to AR AIDS Res Hum Retroviruses 2007 12 335 40 10.1089/aid.2006.0183 17331042

[B25] Doualla-Bell F Avalos A Gaolathe T Mine M Gaseitsiwe S Ndwapi N Novitsky VA Brenner B Oliveira M Moisi D Moffat H Thior I Essex M Wainberg MA Impact of human immunodeficiency virus type 1 subtype C on drug resistance mutations in patients from Botswana failing a nelfinavir-containing regimen Antimicrob Agents Chemother 2006 12 2210 3 10.1128/AAC.01447-05 16723586 PMC1479146

[B26] Doualla-Bell F Avalos A Brenner B Gaolathe T Mine M Gaseitsiwe S Oliveira M Moisi D Ndwapi N Moffat H Essex M Wainberg MA High prevalence of the K65R mutation in human immunodeficiency virus type 1 subtype C isolates from infected patients in Botswana treated with didanosine-based regimens Antimicrob Agents Chemother 2006 12 4182 5 10.1128/AAC.00714-06 17015626 PMC1693987

[B27] Flandre P Marcelin AG Masquelier B Descamps D Izopet J Charpentier C Aloui C Peytavin G Lavignon M Calvez V Impact of HIV-1 subtype in selecting mutations associated with response to boosted tipranavir in HIV-1-infected protease inhibitor experienced patients [abstract] Antivir Ther 2007 12 Suppl 1 s83

[B28] Grossman Z Vardinon N Chemtob D Alkan ML Bentwich Z Burke M Gottesman G Istomin V Levi I Maayan S Shahar E Schapiro JM Genotypic variation of HIV-1 reverse transcriptase and protease: comparative analysis of clade C and clade B AIDS 2001 12 1453 60 10.1097/00002030-200108170-00001 11504976

[B29] Grossman Z Istomin V Averbuch D Lorber M Risenberg K Levi I Chowers M Burke M Bar Yaacov N Schapiro JM Genetic variation at NNRTI resistance-associated positions in patients infected with HIV-1 subtype C AIDS 2004 12 909 15 10.1097/00002030-200404090-00008 15060438

[B30] Grossman Z Paxinos EE Averbuch D Maayan S Parkin NT Engelhard D Lorber M Istomin V Shaked Y Mendelson E Ram D Petropoulos CJ Schapiro JM Mutation D30N is not preferentially selected by human immunodeficiency virus type 1 subtype C in the development of resistance to nelfinavir Antimicrob Agents Chemother 2004 12 2159 65 10.1128/AAC.48.6.2159-2165.2004 15155216 PMC415604

[B31] Grossman Z Lorber M Thibaut L Shahar E Torten D Levy I Riesenberg K Chowers M Istomin V Averbuch D Kra-Oz Z Pollack S Maayan S Faudon J Schapiro J Virological Response and Resistance to Lopinavir/Ritonavir in Subtype-C Patients12th Conference on Retroviruses and Opportunistic Infections: Boston, MA 2005

[B32] Gupta RK Chrystie IL O'Shea S Mullen JE Kulasegaram R Tong CY K65R and Y181C are less prevalent in HAART-experienced HIV-1 subtype A patients AIDS 2005 12 1916 9 10.1097/01.aids.0000189860.36688.e5 16227803

[B33] Hosseinipour M van Oosterhout JJ Weigel R Nelson J Fiscus S Eron J Kumwenda J Resistance profile of patients failing first line ART in Malawi when using clinical and immunologic monitoring AIDS 2008 – XVII International AIDS Conference: Mexico City, Mexico 2008

[B34] Hsu LY Subramaniam R Bacheler L Paton NI Characterization of mutations in CRF01_AE virus isolates from antiretroviral treatment-naive and -experienced patients in Singapore J Acquir Immune Defic Syndr 2005 12 5 13 10.1097/00126334-200501010-00002 15608517

[B35] Jiang S Xing H Si X Wang Y Shao Y Polymorphism of the protease and reverse transcriptase and drug resistance mutation patterns of HIV-1 subtype B prevailing in China J Acquir Immune Defic Syndr 2006 12 512 4 10.1097/01.qai.0000221688.69596.ba 16810118

[B36] Kandathil AJ Kannangai R Abraham OC Sudarsanam TD Pulimood SA Sridharan G Genotypic resistance profile of HIV-1 protease gene: a preliminary report from Vellore, south India Indian J Med Microbiol 2008 12 151 4 10.4103/0255-0857.40530 18445952

[B37] Kantor R Shafer RW Mutetwa S Zijenah L Johnston E Lloyd R Von Lieven A Israelski D Katzenstein DA HIV-1 subtype C reverse transcriptase and protease genotypes in patients from Zimbabwe failing antiretroviral therapy [abstract] Abstracts of the Interscience Conference on Antimicrobial Agents & Chemotherapy 2002 12 293

[B38] Kantor R Katzenstein DA Efron B Carvalho AP Wynhoven B Cane P Clarke J Sirivichayakul S Soares MA Snoeck J Pillay C Rudich H Rodrigues R Holguin A Ariyoshi K Bouzas MB Cahn P Sugiura W Soriano V Brigido LF Grossman Z Morris L Vandamme AM Tanuri A Phanuphak P Weber JN Pillay D Harrigan PR Camacho R Schapiro JM Shafer RW Impact of HIV-1 subtype and antiretroviral therapy on protease and reverse transcriptase genotype: results of a global collaboration PLoS Med 2005 12 e112 10.1371/journal.pmed.0020112 15839752 PMC1087220

[B39] Lolekha R Sirivichayakul S Siangphoe U Pancharoen C Kaewchana S Apateerapong W Mahanontharit A Chotpitayasunondh T Ruxrungtham K Phanuphak P Ananworanich J Resistance to dual nucleoside reverse-transcriptase inhibitors in children infected with HIV clade A/E Clin Infect Dis 2005 12 309 12 10.1086/427026 15655753

[B40] Machado ES Lambert JS Watson DC Afonso AO Da Cunha SM Nogueira SA Caride E Oliveira RH Sill AM DeVico A Tanuri A Genotypic resistance and HIV-1 subtype in Brazilian children on dual and triple combination therapy J Clin Virol 2004 12 24 31 10.1016/j.jcv.2003.08.001 15072750

[B41] Marconi VC Sunpath H Lu Z Gordon M Koranteng-Apeagyei K Hampton J Carpenter S Giddy J Ross D Holst H Losina E Walker BD Kuritzkes DR Prevalence of HIV-1 drug resistance after failure of a first highly active antiretroviral therapy regimen in KwaZulu Natal, South Africa Clin Infect Dis 2008 12 1589 97 10.1086/587109 18419495 PMC2692213

[B42] Nadembega WM Giannella S Simpore J Ceccherini-Silberstein F Pietra V Bertoli A Pignatelli S Bellocchi MC Nikiema JB Cappelli G Bere A Colizzi V Perno CP Musumeci S Characterization of drug-resistance mutations in HIV-1 isolates from non-HAART and HAART treated patients in Burkina Faso J Med Virol 2006 12 1385 91 10.1002/jmv.20709 16998878

[B43] Novitsky V Wester CW DeGruttola V Bussmann H Gaseitsiwe S Thomas A Moyo S Musonda R Van Widenfelt E Marlink RG Essex M The reverse transcriptase 67N 70R 215Y genotype is the predominant TAM pathway associated with virologic failure among HIV type 1C-infected adults treated with ZDV/ddI-containing HAART in southern Africa AIDS Res Hum Retroviruses 2007 12 868 78 10.1089/aid.2006.0298 17678469

[B44] Richard N Juntilla M Abraha A Demers K Paxinos E Galovich J Petropoulos C Whalen CC Kyeyune F Atwine D Kityo C Mugyenyi P Arts EJ High prevalence of antiretroviral resistance in treated Ugandans infected with non-subtype B human immunodeficiency virus type 1 AIDS Research & Human Retroviruses 2004 12 355 64 10.1089/088922204323048104 15157354

[B45] Ruibal-Brunet IJ Cuevas MT Diaz-Torres H Villahermosa ML Noa-Romero E Vazquez de Parga E Blanco de Armas M Perez-Alvarez L Genotypic resistance mutations to antiretroviral drugs in HIV-1 B and non-B subtypes from Cuba Rev Panam Salud Publica 2001 12 174 80 10.1590/S1020-49892001000900005 11702373

[B46] Sen S Tripathy SP Patil AA Chimanpure VM Paranjape RS High prevalence of human immunodeficiency virus type 1 drug resistance mutations in antiretroviral treatment-experienced patients from Pune, India AIDS Res Hum Retroviruses 2007 12 1303 8 10.1089/aid.2007.0090 17961120

[B47] Sirivichayakul S Nucleoside analogue mutations and Q151M in HIV-1 subtype A/E infection treated with nucleoside reverse transcriptase inhibitors AIDS 2003 12 1889 96 10.1097/00002030-200309050-00007 12960821

[B48] Soares EA Santos AF Sousa TM Sprinz E Martinez AM Silveira J Tanuri A Soares MA Differential drug resistance acquisition in HIV-1 of subtypes B and C PLoS ONE 2007 12 e730 10.1371/journal.pone.0000730 17710130 PMC1939879

[B49] Solomon S Balakrishnan P Shetty N Cecelia A Madhavan V Ganesh A Kumarasamy N Celentano D Solomon S Gallant J Prevalence of Treatment Failure and Drug Resistance among Treatment-experienced HIV-1-infected Individuals at a Tertiary HIV Referral Center in South India14th Conference on Retroviruses and Opportunistic Infections: Los Angeles, CA 2007

[B50] Sunpath H France H Tarin M Marconi VC Murphy R Kanegai C Lu Z Losina E Walker BD Kuritzkes D Prospective analysis of HIV-1 Drug Resistance After Virologic Failure on Antiretroviral Therapy (ART): Initial Results from a Paediatric Cohort Study from KZN, South Africa15th Conference on Retrovirus and Opportunistic Infections: Boston, MA 2008

[B51] Sukasem C Churdboonchart V Sukeepaisarncharoen W Piroj W Inwisai T Tiensuwan M Chantratita W Genotypic resistance profiles in antiretroviral-naive HIV-1 infections before and after initiation of first-line HAART: impact of polymorphism on resistance to therapy Int J Antimicrob Agents 2008 12 277 81 18182278 10.1016/j.ijantimicag.2007.10.029

[B52] Tebit D Makamtse A Yameogo S Sangare L Kraeusslich H-G Characterization of HIV drug resistance mutations among antiretroviral drug-exposed subjects in Burkina Faso AIDS 2006 – XVI International AIDS Conference: Toronto, Canada 2006

[B53] Tebit DM Sangare L Makamtse A Yameogo S Somlare H Bado G Kouldiaty BG Sathiandee K Tiba F Sanou I Ouedraogo-Traore R Zoungrana L Diallo I Drabo JY Krausslich HG HIV drug resistance pattern among HAART-exposed patients with suboptimal virological response in Ouagadougou, Burkina Faso J Acquir Immune Defic Syndr 2008 12 17 25 10.1097/QAI.0b013e318182d2bc 18667925

[B54] Vergne L Kane CT Laurent C Diakhate N Gueye NF Gueye PM Sow PS Faye MA Liegeois F Ndir A Laniece I Peeters M Ndoye I Mboup S Delaporte E Low rate of genotypic HIV-1 drug-resistant strains in the Senegalese government initiative of access to antiretroviral therapy AIDS 2003 12 s31 s38 14565607 10.1097/00002030-200317003-00005

[B55] Waleria-Aleixo A Martins AN Arruda MB Brindeiro RM Da-Silva RM Nobre FF Greco DB Tanuri A Drug resistance mutation (DRM) profile and accumulation kinetic in HIV+ individuals infected with subtype B and F failing highly active antiretroviral therapy (HAART) is influenced by different viral codon usage Antimicrob Agents Chemother 2008 12 4497 502 10.1128/AAC.00820-08 18838582 PMC2592894

[B56] Wallis C Bell C Boulme R Sanne I Venter F Papathanasopoulos M Stevens W Emerging ART Drug Resistance in Subtype C: Experience from the 2 Clinics in Johannesburg, South Africa14th Conference of Retroviruses and Opportunistic Infections: Los Angeles, CA 2007

[B57] Weidle PJ Downing R Sozi C Mwebaze R Rukundo G Malamba S Respess R Hertogs K Larder B Ochola D Mermin J Samb B Lackritz E Development of phenotypic and genotypic resistance to antiretroviral therapy in the UNAIDS HIV Drug Access Initiative – Uganda AIDS 2003 12 Suppl 3 s39 s48 14565608 10.1097/00002030-200317003-00006

[B58] Welz T Easterbrook P UK Collaborative Group on HIV Drug Resistance Impact of HIV-1 subtype on genotypic resistance to protease inhibitors in the UK13th Conference on Retroviruses and Opportunistic Infections: Denver, CO 2006

[B59] Abecasis AB Deforche K Snoeck J Bacheler LT McKenna P Carvalho AP Gomes P Camacho RJ Vandamme AM Protease mutation M89I/V is linked to therapy failure in patients infected with the HIV-1 non-B subtypes C, F or G AIDS 2005 12 1799 806 10.1097/01.aids.0000188422.95162.b7 16227787

[B60] Papa A Papadimitriou E Papoutsi A Malissiovas N Kiosses VG Antoniadis A Genetic variation of the protease and reverse transcriptase genes in HIV-1 CRF04_cpx strains AIDS Res Hum Retroviruses 2002 12 677 80 10.1089/088922202760019392 12079565

[B61] Quarleri JF Rubio A Carobene M Turk G Vignoles M Harrigan RP Montaner JSG Salomon H Gomez-Carrillo M HIV type 1 BF recombinant strains exhibit different pol gene mosaic patterns: Descriptive analysis from 284 patients under treatment failure AIDS Res Hum Retroviruses 2004 12 1100 7 10.1089/aid.2004.20.1100 15585101

[B62] Gonzales MJ Wu TD Taylor J Belitskaya I Kantor R Israelski D Chou S Zolopa AR Fessel WJ Shafer RW Extended spectrum of HIV-1 reverse transcriptase mutations in patients receiving multiple nucleoside analog inhibitors AIDS 2003 12 791 9 10.1097/00002030-200304110-00003 12660525 PMC2573403

[B63] Coutsinos D Invernizzi CF Xu H Moisi D Oliveira M Brenner BG Wainberg MA Template usage is responsible for the preferential acquisition of the K65R reverse transcriptase mutation in subtype C variants of human immunodeficiency virus type 1 J Virol 2009 12 2029 33 10.1128/JVI.01349-08 19073730 PMC2643749

[B64] Xu HT Martinez-Cajas JL Ntemgwa ML Coutsinos D Frankel FA Brenner BG Wainberg MA Effects of the K65R and K65R/M184V reverse transcriptase mutations in subtype C HIV on enzyme function and drug resistance Retrovirology 2009 12 14 19210791 10.1186/1742-4690-6-14 PMC2644664

[B65] Bracciale L Di Giambenedetto S Colafigli M La Torre G Prosperi M Santangelo R Marchetti S Cauda R Fadda G De Luca A Virological suppression reduces clinical progression in patients with multiclass-resistant HIV type 1 AIDS Res Hum Retroviruses 2009 12 261 7 10.1089/aid.2008.0136 19292594

[B66] Di Giambenedetto S Colafigli M Pinnetti C Bacarelli A Cingolani A Tamburrini E Cauda R De Luca A Genotypic resistance profile and clinical progression of treatment-experienced HIV type 1-infected patients with virological failure AIDS Res Hum Retroviruses 2008 12 149 54 10.1089/aid.2007.0070 18240962

[B67] Zaccarelli M Tozzi V Lorenzini P Trotta MP Forbici F Visco-Comandini U Gori C Narciso P Perno CF Antinori A Multiple drug class-wide resistance associated with poorer survival after treatment failure in a cohort of HIV-infected patients AIDS 2005 12 1081 9 10.1097/01.aids.0000174455.01369.ad 15958840

[B68] World Health Organization Antiretroviral Therapy for HIV infection in adults and adolescents: Recommendations for a public health approach 2006 http://www.who.int/hiv/pub/arv/adult/en/index.html 23741771

[B69] DHHS Panel on Antiretroviral Guidelines for Adults and Adolescents Guidelines for the Use of Antiretroviral Agents in HIV-1-Infected Adults and Adolescents 2008 http://aidsinfo.nih.gov/contentfiles/AdultandAdolescentGL.pdf

[B70] Panel de expertos de Gesida y Plan Nacional sobre el Sida Recomendaciones de Gesida/Plan Nacional sobre el Sida respecto al tratamiento antirretroviral en adultos infectados por el virus de la inmunodeficiencia humana (Actualización enero de 2008) 2008 http://www.gesida.seimc.org/index.asp 10.1016/j.eimc.2013.04.009 24161378

[B71] Nachega JB Hislop M Dowdy DW Chaisson RE Regensberg L Maartens G Adherence to nonnucleoside reverse transcriptase inhibitor-based HIV therapy and virologic outcomes Ann Intern Med 2007 12 564 73 17438315 10.7326/0003-4819-146-8-200704170-00007

[B72] Poonpiriya V Sungkanuparph S Leechanachai P Pasomsub E Watitpun C Chunhakan S Chantratita W A study of seven rule-based algorithms for the interpretation of HIV-1 genotypic resistance data in Thailand J Virol Methods 2008 12 79 86 10.1016/j.jviromet.2008.03.017 18462814

[B73] Marcelin AG Masquelier B Descamps D Izopet J Charpentier C Alloui C Bouvier-Alias M Signori-Schmuck A Montes B Chaix ML Amiel C Santos GD Ruffault A Barin F Peytavin G Lavignon M Flandre P Calvez V Tipranavir-ritonavir genotypic resistance score in protease inhibitor-experienced patients Antimicrob Agents Chemother 2008 12 3237 43 10.1128/AAC.00133-08 18625773 PMC2533483

[B74] Vergne L Snoeck J Aghokeng A Maes B Valea D Delaporte E Vandamme AM Peeters M Van Laethem K Genotypic drug resistance interpretation algorithms display high levels of discordance when applied to non-B strains from HIV-1 naive and treated patients FEMS Immunol Med Microbiol 2006 12 53 62 10.1111/j.1574-695X.2005.00011.x 16420597

[B75] Snoeck J Kantor R Shafer RW Van Laethem K Deforche K Carvalho AP Wynhoven B Soares MA Cane P Clarke J Pillay C Sirivichayakul S Ariyoshi K Holguin A Rudich H Rodrigues R Bouzas MB Brun-Vezinet F Reid C Cahn P Brigido LF Grossman Z Soriano V Sugiura W Phanuphak P Morris L Weber J Pillay D Tanuri A Harrigan RP Camacho R Schapiro JM Katzenstein D Vandamme AM Discordances between interpretation algorithms for genotypic resistance to protease and reverse transcriptase inhibitors of human immunodeficiency virus are subtype dependent Antimicrob Agents Chemother 2006 12 694 701 10.1128/AAC.50.2.694-701.2006 16436728 PMC1366873

[B76] Champenois K Bocket L Deuffic-Burban S Cotte L Andre P Choisy P Yazdanpanah Y Expected response to protease inhibitors of HIV-1 non-B subtype viruses according to resistance algorithms AIDS 2008 12 1087 9 10.1097/QAD.0b013e3282ff629b 18520355

