# Supplementary material for: Differences in resistance mutations among HIV-1 non-subtype B infections: a systematic review of evidence (1996–2008)
Source: J Int AIDS Soc. 2009 Jun 30;12:11. doi: 10.1186/1758-2652-12-11 (PMC2713201; doi:10.1186/1758-2652-12-11)
Supplement: Additional file 1 — Table S1. Quality score of reviewed studies. [file 1758-2652-12-11-S1.doc]

| Publications | Number of ART treated patients with non-B infection | Research question(s) explicit and clear | First therapeutic failure of studied drug or drug combination | Mutations grouped by drug or relevant drug combination used | Sample is representative | Sample protocol guided | Compared to B or other non-B subtype | Compared to same subtype consensus or wild type sequence database | Compared to genotype of same virus before therapy | Type of publication | Total |
| --- | --- | --- | --- | --- | --- | --- | --- | --- | --- | --- | --- |
| Abecasis AB, 2005 | 173 | 1 | 0 | 0 | 1 | 0 | 1 | 1 | 0 | Journal article | 4 |
| Ariyoshi K, 2003 | 216 | 1 | 0 | 0 | 1 | 0 | 1 | 1 | 0 | Journal article | 4 |
| Barth RE, 2008 | 31 | 1 | 1 | 1 | 0 | 1 | 1 | 1 | 0 | Brief report | 6 |
| Calazans A, 2005 | 22 | 1 | 0 | 1 | 1 | 1 | 1 | 1 | 0 | Journal article | 6 |
| Camacho R, 2005 | 240 | 1 | 0 | 0 | 1 | 1 | 1 | 1 | 0 | Conference abstract | 5 |
| Cane PA, 2001 | 30 | 1 | 0 | 0 | 1 | 0 | 0 | 1 | 0 | Journal article | 3 |
| Cavalcanti AM, 2007 | 576 | 1 | 0 | 0 | 1 | 0 | 1 | 1 | 0 | Journal article | 4 |
| Chaix ML, 2005 | 38 | 1 | 1 | 1 | 1 | 1 | 1 | 1 | 0 | Journal article | 7 |
| Couto-Fernandez JC, 2005 | 488 | 1 | 0 | 0 | 1 | 0 | 1 | 1 | 0 | Journal article | 4 |
| De Sa-Filho DJ, 2007 | 83 | 1 | 0 | 0 | 0 | 0 | 1 | 0 | 0 | Journal article | 2 |
| Deshpande A, 2007 | 223 | 1 | 1 | 1 | 0 | 1 | 1 | 1 | 0 | Journal article | 6 |
| Doualla-Bell F, 2006 | 16 | 1 | 1 | 1 | 1 | 0 | 1 | 1 | 0 | Journal article | 6 |
| Doualla-Bell F, 2006 | 23 | 1 | 0 | 0 | 1 | 1 | 0 | 1 | 0 | Journal article | 4 |
| Dumans AT, 2004 | N/A | N/A |  |  |  |  |  |  |  |  | NA |
| Flandre P, 2007 | 143 | 1 | 0 | 1 | 0 | 1 | 1 | 1 | 0 | Conference abstract | 5 |
| Grossman Z, 2001 | 133 | 1 | 0 | 0 | 1 | 1 | 1 | 1 | 0 | Journal article | 5 |
| Grossman Z, 2004 nfv | 491 | 1 | 1 | 1 | 1 | 1 | 1 | 1 | 0 | Journal article | 7 |
| Grossman Z, 2005 | 224 | 1 | 1 | 1 | 1 | 0 | 1 | 0 | 0 | Abstract | 5 |
| Grossman Z, 2004 | 84 | 1 | 1 | 1 | 1 | 0 | 1 | 1 | 0 | Journal article | 6 |
| Gupta RK, 2005 | 472 | 1 | 0 | 0 | 1 | 0 | 1 | 0 | 0 | Research letter | 3 |
| Hosseinipour MC, 2008 | 101 | 1 | 1 | 1 | 1 | 1 | 1 | 0 | 0 | Conference paper | 6 |
| Hsu LY, 2005 | 79 | 1 | 0 | 0 | 1 | 1 | 1 | 1 | 0 | Journal article | 5 |
| Jiang S, 2006 | 126 | 1 | 0 | 0 | 1 | 0 | 1 | 1 | 0 | Research letter | 4 |
| Kandathil AJ, 2008 | 3 | 1 | 0 | 0 | 0 | 0 | 1 | 0 | 0 | Journal article | 2 |
| Kantor R, 2005 | 3686 | 1 | 0 | 0 | 1 | 1 | 1 | 1 | 0 | Journal article | 5 |
| Kantor R, 2002 | 21 | 1 | 1 | 0 | 1 | 0 | 1 | 1 | 0 | Journal article | 5 |
| Lolekha R, 2005 | 100 | 1 | 1 | 1 | 1 | 1 | 1 | 0 | 0 | Brief report | 6 |
| Machado ES, 2004 | 37 | 1 | 0 | 0 | 1 | 1 | 1 | 0 | 0 | Journal article | 4 |
| Marconi VC, 2008 | 147 | 1 | 0 | 1 | 1 | 1 | 0 | 1 | 0 | Journal article | 5 |
| Nadembega WM, 2006 | 16 | 1 | 0 | 0 | 1 | 0 | 0 | 1 | 0 | Journal article | 3 |
| Novitsky V, 2007 | 23 | 1 | 1 | 1 | 1 | 1 | 1 | 1 | 0 | Research article | 7 |
| Papa A, 2007 | 5 | 1 | 0 | 0 | 1 | 0 | 1 | 0 | 0 | Journal article | 3 |
| Quarleri JF, 2004 | 284 | 1 | 0 | 0 | 1 | 1 | 1 | 1 | 0 | Journal article | 5 |
| Richard N, 2004 | 59 | 1 | 1 | 1 | 1 | 1 | 1 | 0 | 0 | Journal article | 6 |
| Ruibal-Brunet IJ, 2001 | 22 | 1 | 0 | 0 | 1 | 1 | 1 | 0 | 0 | Journal article | 4 |
| Sen S, 2007 | 33 | 1 | 0 | 0 | 1 | 0 | 0 | 1 | 1 | Journal article | 4 |
| Sirivichayakul S, 2003 | 43 | 1 | 0 | 1 | 1 | 1 | 0 | 0 | 0 | Journal article | 4 |
| Soares EA, 2007 | 57 | 1 | 0 | 0 | 1 | 1 | 1 | 0 | 0 | Journal article | 4 |
| Solomon S, 2008 | 95 | 1 | 0 | 0 | 0 | 1 | 0 | 0 | 0 | Conference poster | 2 |
| Susakem C, 2008 | 43 | 1 | 0 | 0 | 1 | 1 | 1 | 1 | 0 | Journal article | 5 |
| Sunpath H, 2008 | 278 | 1 | 0 | 0 | 1 | 1 | 0 | 0 | 0 | Conference paper | 3 |
| Sylla M, 2008 | 113 | 1 | 1 | 1 | 0 | 1 | 1 | 1 | 0 | Journal article | 6 |
| Tebit D, 2008 | 87 | 1 | 0 | 0 | 1 | 1 | 1 | 1 | 0 | Journal article | 5 |
| Tebit D, 2006 | 55 | 1 | 0 | 0 | 0 | 1 | 0 | 0 | 0 | Conference abstract | 2 |
| Tupinambas U, 2005 | 55 | 1 | 1 | 1 | 1 | 1 | 1 | 1 | 0 | Journal article | 7 |
| Vergne L, 2003 | 80 | 1 | 0 | 0 | 1 | 1 | 1 | 0 | 1 | Research letter | 5 |
| Waleria-Aleixo A, 2008 | 238 | 1 | 0 | 0 | 1 | 0 | 1 | 1 | 0 | Journal article | 4 |
| Wallis C, 2007 | 115 | 1 | 0 | 0 | 1 | 1 | 1 | 0 | 0 | Conference poster | 4 |
| Weidle PJ, 2003 | 116 | 1 | 1 | 1 | 1 | 1 | 1 | 1 | 0 | Journal article | 7 |
| Welz T, 2006 | 1155 | 1 | 0 | 0 | 1 | 1 | 1 | 1 | 0 | Conference poster | 5 |
