# Supplementary material for: Differences in resistance mutations among HIV-1 non-subtype B infections: a systematic review of evidence (1996–2008)
Source: J Int AIDS Soc. 2009 Jun 30;12:11. doi: 10.1186/1758-2652-12-11 (PMC2713201; doi:10.1186/1758-2652-12-11)
Supplement: Additional file 2 — Table S2. Characteristics of the studies evaluated. [file 1758-2652-12-11-S2.doc]

| Reference | Study | Study design | Algorithm/list used to define resistance mutations | Publication type |
| --- | --- | --- | --- | --- |
| 59 | Abecasis AB, 2005 Portugal | Cross-sectional | NA | Journal article |
| 17 | Ariyoshi K, 2003 Japan | Cross-sectional | All positions evaluated | Journal article |
| 16 | Barth RE, 2008 South Africa | Longitudinal | IAS | Brief report |
| 18 | Calazans A, 2005 Brazil | Cross-sectional | NA | Journal article |
| 19 | Camacho R, 2005  Portugal | Cross-sectional | NR | Conference abstract |
| 20 | Cane PA, 2001 United Kingdom | Cross-sectional | Stanford database | Journal article |
| 15 | Cavalcanti AM, 2007 Brazil | Cross-sectional | RENAGENO | Journal article |
| 21 | Chaix ML, 2005 Cote d’Ivoire | Longitudinal cohort | ANRS | Journal article |
| 22 | Couto-Fernandez JC, 2005 Brazil | Cross-sectional | IAS-US | Journal article |
| 23 | De Sa-Filho, DJ 2007 Brazil | Cross-sectional | Stanford database | Journal article |
| 24 | Deshpande A, 2007 India | Cross-sectional | ANRS, IAS and Stanford database | Journal article |
| 25 | Doualla-Bell,F 2006 Botswana | Retrospective cohort study | NR | Journal article |
| 26 | Doualla-Bell F, 2006 Botswana | Cross-sectional | NR | Journal article |
| 13 | Dumans AT, 2004 Brazil | Cross-sectional | IAS-US | Journal article |
| 27 | Flandre P, 2007 | Longitudinal cohort | Defined by study | Conference abstract |
| 28 | Grossman Z, 2001 Israel | Cross-sectional | NR | Journal article |
| 29 | Grossman Z, 2004 Israel | Cross-sectional | Positions related to resistance in B | Journal article |
| 30 | Grossman Z, 2004 Israel | Cross-sectional | Stanford database | Journal article |
| 31 | Grossman Z, 2005 Israel | Cross-sectional | Stanford database | Abstract |
| 32 | Gupta RK, 2005 United Kingdom | Cross-sectional | NR | Journal article |
| 33 | Hosseinipour M, 2008 Malawi | Cross-sectional | Trugene, IAS-USA list | Conference paper |
| 34 | Hsu, LY, 2005 Singapore | Cross-sectional | Present in subtype B-based resistance algorithm or statistical significance found | Journal article |
| 35 | Jiang S, 2006 China | Cross-sectional | All positions evaluated | Journal article |
| 36 | Kandathil AJ, 2008 | Cross-sectional | Stanford | Journal article |
| 37 | Kantor R, 2002 Zimbabwe | Cross-sectional | NR | Journal article |
| 38 | Kantor R, 2005 International | Cross-sectional | NR | Journal article |
| 39 | Lolekha R, 2005 Thailand | Cross-sectional | IAS USA list. E44D/V118I was not included as resistance mutations | Journal article |
| 40 | Machado ES, 2004 Brazil | Cross-sectional | Stanford database | Journal article |
| 41 | Marconi VC, 2008 South Africa | Cross-sectional | Study protocol | Journal article |
| 42 | Nadembega WM, 2006 Burkina Faso | Cross-sectional study | Stanford database | Journal article |
| 43 | Novitsky V, 2007 Bostwana | Clinical trial | Stanford database | Journal article |
| 60 | Papa A, 2002 Greece | Cross-sectional | NR | Journal article |
| 61 | Quarleri JF, 2004 Argentina | Cross-sectional | NR | Journal article |
| 44 | Richard N, 2004 Uganda | Cross-sectional and longitudinal components | NR | Journal article |
| 45 | Ruibal-Brunet IJ, 2001 Cuba | Cross-sectional | IAS-US | Journal article |
| 46 | Sen S, 2007 India | Retrospective cohort | IAS-US | Journal article |
| 47 | Sirivichayakul S, 2003 Thailand | Clinical trial | NR | Journal article |
| 48 | Soares EA, 2007 Brazil | Retrospective cohort | IAS-US | Journal article |
| 49 | Solomon S, 2007 India | Cross-sectional | Stanford | Conference poster |
| 50 | Sunpath H, 2008 South Africa | Cross-sectional | NR |  |
| 51 | Sukasem C, 2008 | Cross-sectional | Stanford | Journal article |
| 14 | Sylla M, 2008 Burkina Faso | Cross-sectional | Stanford | Journal article |
| 52 | Tebit D, 2006 Burkina Faso | Cross-sectional | NR | Abstract |
| 53 | Tebit DM, 2008 Burkina Faso | Cross-sectional | Stanford Database | Journal article |
| 12 | Tupinambas U, 2005 Brazil | Cross-sectional | IAS-US and RENAGENO | Journal article |
| 54 | Vergne L, 2003 Senegal | Cross-sectional and longitudinal components | Stanford Database | Journal article |
| 55 | Waleria-Aleixo A, 2008 | Cross-sectional | RENAGENO | Journal article |
| 56 | Wallis C, 2007 South Africa | Cross-sectional | Stanford | Conference poster |
| 57 | Weidle PJ, 2003 Uganda | Cross-sectional | Consensus subtype B reference | Journal article |
| 58 | Welz T, 2006 United Kingdom | Cross-sectional | Defined by study | Conference poster |

NR: non reported

IAS US: International AIDS Society USA list of resistance mutations (USA)

ANRS: Algorithm from Agence Nationale de recherche sur le SIDA et les hépatites virales (France)

RENAGENO : Algorithm from Rede Nacional de Laboratórios de Genotipagem (Brazil)

RT: HIV reverse transcriptase

PR: HIV protease

Note: Recombinant forms are referred to using short abbreviations not inclusive of the term CRFnn_ in order to conserve space (e.g., CRF01_AE is recorded as AE).
