# Supplementary material for: Differences in resistance mutations among HIV-1 non-subtype B infections: a systematic review of evidence (1996–2008)
Source: J Int AIDS Soc. 2009 Jun 30;12:11. doi: 10.1186/1758-2652-12-11 (PMC2713201; doi:10.1186/1758-2652-12-11)
Supplement: Additional File 3 — Table S3. Major differences in subtype B versus non-B resistance genotype patterns in patients on ART. [file 1758-2652-12-11-S3.doc]

| **Study, year, place** | **Subtype, number of isolates, and number of patients** | **Subtypes compared** | **Mutations with possible role in ART resistance** | **Differences in mutation frequency (RT)** | **Differences in mutation frequency (PR)** | **Other findings** |
| --- | --- | --- | --- | --- | --- | --- |
| Abecasis AB, 2005 Portugal | A ( 23), B (438), C(69), D (15), F (26), G (341), H(19), J (10), and K (13)  173 PI treated patients. The distribution by drug and subtype was not reported | G exposed to PIs vs naïve G | M89I/V | NA |  | Mutations mainly occur in presence of major PI resistance mutations |
|  |  | C and F exposed to PIs vs naïve C and F isolates | M89I | NA |  | Mutations mainly occur in presence of major PI resistance mutations |
| Ariyoshi K, 2003 Japan | 216 patients failing ARV. Subtype B (162 sequences), subtype AE (45 sequences) from patients failing ART, including ZDV, 3TC, d4T, ddI, NVP, EFV, NFV, IDV, SQV, RTV | AE vs B | RT mutation G196E in AE was associated with NRTI exposure | T69N and the V75M were seen more commonly in AE than in subtype B. V75M significantly associated with d4T in AE but not subtype B | L10F, K20I, L33I, N88S were more commonly seen in AE. D30N, 71V, N88D exclusively seen in subtype B | 11 AE infected patients were failing NFV, but D30N not seen |
| Barth RE, 2008 South Africa | 31 patients failing a combination of 1thymidine analog + 3TC and 1 NNRTI as first line therapy. 23 samples available | C |  | No TAMs or K65R observed. 58% and 26% had at least two and three NNRTI major mutations respectively. This was probably related to high (21%) frequency of NNRTI mutations at baseline |  |  |
| Calazans A, 2005 Brazil | F (22) from NFV or SQV failing patients | F vs B |  | NA | 1/22 patients with subtype F developed L90M vs 13/54 patients with subtype B |  |
| Camacho R, 2005 Portugal | B (183) and G (57) carrying mutations at position 82 | B vs G | 82M |  | Mutation V82M only found in G subtype |  |
| Cane PA, 2001 United Kingdom | 28 ART-naïve patients and 30 ART-treated patients who took PIs for subtype C HIV-1 infection | C |  | NA | 1 of 8 patients failing NFV had D30N. The others had L90M and other minor mutations |  |
| Cavalcanti AM, 2007 Brazil | 576 samples from patients failing therapy: 413 B and 60 F | B vs F |  | NRTI-associated mutations occurred most frequently in codon 211, in subtype F; and in codon 210 in subtype B | Mutations in codons 35, 36 were present only in subtype F and in codon 63 only in subtype B | K65R seen in 3.4% of cases |
| Chaix ML, 2005 Cote d’Ivoire | 38 Children failing ART with 2 NRTIs + NFV or 1 NNRTI. CRF02-AG (36) | AG only |  | No significant difference | No D30N mutation found in AG isolates exposed to NFV |  |
| Couto-Fernandez JC, 2005 Brazil | Total 488 samples. 445 subtype B, 24 subtype F and 16 BF recombinants | B vs (F and BF) |  | No significant difference | L63P more frequent in B and 36I more frequent in non-Bs. |  |
| De Sa-Filho DJ, 2007 Brazil | 83 patients failing NRTI, NNRTI and PI therapy | B, F, C |  | No difference found | No difference found |  |
| Deshpande A, 2007 India | From 223 patients failing ART (2NRTIs + 1 NNRTI), 112 RT sequences could be amplified. 109 were C subtype | C only | Mutations at positions 203, 208 and 221 highly correlated with ART | The NRTI mutations K65R and Q151M found in 2 patients each. L100I was absent. The NNRTI mutations V106M and A98G/S were present in 10% and 27% of treated patients | NA |  |
| Doualla-Bell F, 2006 Botswana | 16 patients failing NFV-containing regimens Bostwana. All were subtype C | C only | M89I | NA | The prevalence of the D30N mutation was 54%, followed by L90M (31%). The M89I substitution was strongly associated with therapy |  |
| Doualla-Bell F, 2006 Botswana | 23 patients failing first or second line ART which included ddI/d4T or ddI/ZDV or ZDV/3TC or d4T/3TC as NNRTI backbone | C only |  | K65R seen in 30% of patients |  |  |
| Dumans AT, 2004 Brazil | 170, 35, and 43 RT sequences and 102, 29, and 41 PR sequences belonging to subtype B, C, and F1 isolates respectively were obtained from ART-treated individuals | B, C and F |  | Lower rate of L210W and Q151M. Probable higher genetic barrier to acquisition of mutations at this positions in F subtypes compared with B and C subtypes | NA |  |
| Flandre P, 2007 France | 143 ART-experienced patients receiving TPV as part of salvage therapy. 127 B and 16 were non-B | B vs non-B |  |  | The inclusion of non-B subtypes resulted in different scoring for mutations associated with resistance compared to what is obtained if B subtype the only one included |  |
| Grossman Z, 2001 Israel | B (73), C (58), A (2) patients failing ART that included NRTI, NNRTI or PI drug classes alone or in combination | C vs B |  | A98G/S was more frequent in C than in B subtype | Mutations more frequent in C than in B: L10I, M36I. Mutations more frequent in B than in C: D30N, M36I, L63P, A71I, G73I, V77I, I84V, L90M | K65R not reported |
| Grossman Z, 2004 Israel | B (150) and C (341) HIV-1-infected Individuals treated with EFV and NVP used in combination with NRTIs as the first or second regimen | C vs B |  | V106M preferentially selected by EFV in subtype C isolates (prevalence 24%). A98G/S was present as a common polymorphism in C subtype | NA |  |
| Grossman Z, 2004 Israel | C (159) and B (65) from patients failing first PI therapy with either NFV or IDV | C vs B |  | NA | After NFV failure, D30N was less frequent in C (7%) than in B (23%), while L90M was similar. Different prevalence of M36I (98 and 36%), L63P (35 and 59%), A71V (3 and 32%), V77I (0 and 36%), and I93L (91 and 32%) were seen in C and B subtypes respectively |  |
| Grossman Z, 2005 Israel | 49 non-B and 35 B samples from patients failing LPV/RTV | B vs non-B |  | NA | No difference in mutations nor frequency of mutations reported |  |
| Gupta RK, 2005, United Kingdom | 472 samples: B (179), A (36), C (60), D (23), AE (14), AG (30), G (6), J (5) from ART failing patients | B vs other non-subtype Bs |  | Subtype A showed a lower propensity to develop K65R and Y181C than in B or C subtypes despite similar exposure to selecting drugs | NA |  |
| Hosseinipour M, 2008 Malawi | 101 patients experiencing therapeutic failure of the first line combination d4T or ZDV/3TC/NVP or EFV | Assumed to be mainly C |  | High frequency of K65R or K70E (23%) mutations among NRTI resistance viruses | NA |  |
| Hsu L-Y, 2005 Singapore | AE (69), B (18), C (2) AE (65) exposed to ≥ 1 NRTIs AE (21) exposed to ≥ 1 NNRTIs AE (34) exposed to PIs | AE only | The PR mutations 74A/S and N83D | Small differences in prevalence of TAM pathways. V106M present in 14% of patients exposed to NNRTIs | Higher frequency of mutations in AE virus than in B virus at positions K20I, L33F, M36I, and G48V. Mutations M46I/L, L63P, A71V, V77I, V82A and I93L were less frequent in AE than in B |  |
| Jiang S, 2006 China | B subtype circulating in China (126) and exposed to NRTIs and/or NNRTIs | Chinese B subtype | H221Y probably associated witn NNRTI rather than NRTI resistance | No other major difference reported | NA |  |
| Kandathil AJ, 2008 | 3 patients failing PI based therapy | C |  |  | No novel mutation detected |  |
| Kantor R, 2002 Zimbabwe | Subtype C (21). 11 failed first ART regimen. 21 had received NRTIs, 18 had received PIs, 5 had received NNRTIs | C | Mutations at RT positions 53, 123 and 174 | No major difference reported | No major difference reported |  |
| Kantor R, 2005 International | A (461), C (1,185), D (331), F(245), G (293), AE (513), AG (618). Multinational sampling | Various  subtypes | PR: subtype C positions 6 and 64; AG position 15; subtype F position 19; subtype A position 37; and AE position 64. RT position 102 was associated with treatment in subtype C but not in subtype B | Multiple differences in mutation frequencies (see text) | Several differences in mutation frequency (see text) | Each of the 55 known subtype B drug-resistance mutations occurred in at least one non-B isolate, and 44 (80%) of these mutations were associated with ART in at least one non-subtype B. Conversely, of 67 mutations found to be associated with ART in at least one non-subtype B, 61 were also associated with ART in subtype B isolates |
| Lolekha R, 2005 Thailand | AE (100). Children treated with dual NRTI therapy for at least 6 months. Regimens used were any of the following: ZDV+ddI, ZDV+3TC, d4T+ddI, d4T+3TC | AE only |  | No major difference reported | NA |  |
| Machado ES, 2004 Brazil | 32 B subtype infected and 5 F or BF infected children | B vs BF and F |  | Higher frequency of D67N (80% × 43*.*1%), K70R (60% × 21*.*5%) in patients infected with subtype F strains when compared to B-subtype | M36I more frequent in non B subtypes 100% in no-Bs compared to 29% in Bs |  |
| Marconi VC, 2008 South Africa | 115 (of 147 eligible) patients failing first ART regimen: either d4T/3TC/NVP or EFV; or ZDV/ddI/LPVrtv | C |  | In this study, M41L emerged together with T215Y (with or without L210W) in samples from 7 patients. K65R emerged in only 3 patients | NA |  |
| Nadembega WM, 2006 Burkina Faso | 16 chronically infected patients receiving ART (2NRTIs+1NNRTI or PI). 6 had genotyping resistance test done. AG, 06_cpx, 09_cpx | AG |  | No major difference reported | Only minor resistance mutations reported |  |
| Novitsky V, 2007 Bostwana | 23 isolates from patients who failed ZDV/ddI containing ART combinations | C |  | The 67N 70R 215Y NRTI resistance combination the dominant genotype. No 41L or 210W or 219Q genotypes were present. K103N was the most common NNRTI mutation followed by 106A/M, 181C, and 190A |  | 1 case with K65R mutation seen |
| Papa A, 2002 Greece | 7 chronically infected patients. CRF04_cpx | CRF04_cpx |  | No major difference reported | No difference found |  |
| Quarleri JF, 2004 Argentina | 587 (299 B, 284BF). ART treated patients undergoing treatment failure | B vs BF |  | Higher frequency of the 74V and 210W mutations in BF pattern III compared to B subtype | Higher frequency of 10I/V, 46L/I and 63noL mutations in mosaic BF pattern III compared to subtype B. |  |
| Richard N, 2004 Uganda | 59 participants taking ART in cross-sectional study and 16 participants in the longitudinal study | A vs D |  | No major difference reported | No difference reported |  |
| Ruibal-Brunet IJ, 2001 Cuba | 81 B and 22 non-B | B vs non-B |  | No major difference reported | No difference reported |  |
| Sen S, 2007 India | 33 patients. Subtype C (32)), A (1) | C only |  | TAMs were predominant. K65R was not seen | NA |  |
| Sirivichayakul S, 2003 Thailand | AE (43). Patients from HIV-NAT002 d4T/ddI dose reduction study and HIV-NAT003 ZDV/3TC versus ZDV/3TC/ ddI experiencing virologic failure at week 48 and/or week 96 | AE only |  | No major difference reported | NA |  |
| Soares EA , 2007 Brazil | 160 patients. B (84), C (52), F1 (11), D (4). | B vs C |  | Lower rate of accumulation of mutations conferring resistance to ARV in subtype C than in subtype B | Lower rate of accumulation of mutations conferring resistance to ARV in subtype C than in subtype B |  |
| Solomon S, 2007, India | 44 samples from 95 eligible patients who have failed NRTI + NNRTI based regimen | C |  | V106M present in 11% of NNRTI resistance sequences |  | NRTI mutations were not reported |
| Sunpath H, 2008 South Africa | 25 of 278 children who experienced virologic failure to ART 9 were on a ritonavir-based regimen and 16 were on an NNRTI-based regimen | C |  | V106M present in 24% of patients |  | Low prevalence of K65R |
| Sukasem C, 2008 | 43 patients failing PI-based therapy. 29 sequences were AE and 14 were B subtype | B vs AE |  | No difference found | The major mutations D30N, V82A, N88D and L90M conferring resistance to PIs were found in patients infected with the subtype B strain, whereas G48V and I54V were observed  in patients harbouring the CRF01 AE virus. |  |
| Sylla M, 2008 Burkina Faso | Resistance testing done in 46 of 113 patients failing first-line therapy ZDV or d4T/3TC + NVP or EFV | AG vs AGK |  | Subtype K-containing viruses predominantly acquired TAM2 pathway resistance. No patient on d4T developed TAMs |  | Only one of 6 patients who had virologic failure and had no resistance mutations reported non-adherence |
| Tebit D, 2006, Burkina Faso | 55 patients failing ART. AGK (28), AG viruses (23), A (1), B (1) and U (2) | AG and AGK |  | NA | No major difference reported |  |
| Tebit DM, 2008 Burkina Faso | 87 patients failing ARV therapy. 36 (48%) AGK, 30 (40%)  AG | AG, AGK |  | The majority of AGK strains (25 sequences; 69%) carried *≥* 3 TAMs, although this was the case in only 3 (10%) of the AG viruses | Slight variations in frequency of mutations at positions 54, 82 and 90 between AGK and AG | 90% of AGK sequences had resistance to 2 or 3 drug classes, while 52% of AG sequences did so |
| Tupinambas U, 2005 Brazil | 55 NNRTI experienced patients failing NFV-based therapies | B vs BF and F |  | NA | D30N present in 85% of cases and L90M in 15%. The only F isolate had a D30N mutation. The BF recombinant had one isolate with D30N only, one with L90M only and one isolate with both mutations |  |
| Vergne L, 2003 Senegal | 68 naïve and 12 treated with ART | Various subtypes |  | Comparison among groups was not possible | Comparison among groups not possible |  |
| Waleria-Aleixo A, 2008 | 882 samples from patients failing ART. B (639) and F(182), mosaic (56) | B, F |  | RT residues, 210 and 151, have a higher genetic barrier to the acquisition of the resistance codons subtype F1 compared with subtype B |  | The low prevalence of the L210W and Q151M mutations in subtype F1 isolates found highlighted the importance of the differences in codon usage among HIV-1 subtypes |
| Wallis C, 2007 South Africa | 115 patients failing ART from two different clinics. First line: d4T/3TC/NVP or EFV; second line: ZDV/ddI/LPVrtv | C |  | Frequency of K65R was 7 and 15% in patients from two different clinics, respectively. Complex combinations of NNRTI resistance mutation |  |  |
| Weidle PJ, 2003 Uganda | 116 chronically infected adults failing therapy. Subtypes A and D | A vs D |  | No major difference reported | No D30N mutation seen in any of the 3 A subtype isolates included in this study |  |
| Welz T, 2006 United Kingdom | 1143 sequences from PI-exposed individuals from the UK HIV Drug Resistance Database  A (76), AG (16), C (92), B (908), D (42) | A, AG, C, B, D |  |  | Novel PR mutations in non-B subtypes were rare. Such mutations were detected at positions 13, 16, 33, 37, 41, 57, 65, 72, 74 and 89 |  |

[11-60]

NR: not reported

IAS USA: International AIDS Society USA list of resistance mutations (USA)

ANRS: Algorithm of the Agence Nationale de recherche sur le SIDA et les hépatites virales (France)

RENAGENO : Algorithm from Rede Nacional de Laboratórios de Genotipagem (Brazil)

NRTI: nucleside(tide) reverse transcriptase inhibitor; ZDV (zidovudine), d4T (stavudine), ddI (didanosine), 3TC (lamivudine),

NNRTI: non-nucleoside reverse transcriptase inhibitor; EFV (efavirenz), NVP (nevirapine), DLV (delavirdine)

PI: protease inhibitor; NFV (nelfinavir), RTV (ritonavir), IDV (indinavir), LPV (lopinavir), SQV (saquinavir), APV (amprenavir), ATV (atazanavir), TPV (tipranavir)

CRF: circulating recombinant form

Note: Recombinant forms are referred to using short abbreviations not inclusive of the term CRFnn_ in order to conserve space (e.g., CRF01_AE is recorded as AE).
